# An attention-based deep learning model for clinical named entity recognition of Chinese electronic medical records

**DOI:** 10.1186/s12911-019-0933-6

**Published:** 2019-12-05

**Authors:** Luqi Li, Jie Zhao, Li Hou, Yunkai Zhai, Jinming Shi, Fangfang Cui

**Affiliations:** 1 Institute of Medical Information, Chinese Academy of Medical Sciences/Peking Union Medical College, Beijing, China; 2 National Engineering Laboratory for Internet Medical Systems and Applications, The First Affiliated Hospital of Zhengzhou University, Zhengzhou, China

**Keywords:** Named entity recognition, Attention mechanism, Chinese electronic medical records

## Abstract

**Background:**

Clinical named entity recognition (CNER) is important for medical information mining and establishment of high-quality knowledge map. Due to the different text features from natural language and a large number of professional and uncommon clinical terms in Chinese electronic medical records (EMRs), there are still many difficulties in clinical named entity recognition of Chinese EMRs. It is of great importance to eliminate semantic interference and improve the ability of autonomous learning of internal features of the model under the small training corpus.

**Methods:**

From the perspective of deep learning, we integrated the attention mechanism into neural network, and proposed an improved clinical named entity recognition method for Chinese electronic medical records called BiLSTM-Att-CRF, which could capture more useful information of the context and avoid the problem of missing information caused by long-distance factors. In addition, medical dictionaries and part-of-speech (POS) features were also introduced to improve the performance of the model.

**Results:**

Based on China Conference on Knowledge Graph and Semantic Computing (CCKS) 2017 and 2018 Chinese EMRs corpus, our BiLSTM-Att-CRF model finally achieved better performance than other widely-used models without additional features(F1-measure of 85.4% in CCKS 2018, F1-measure of 90.29% in CCKS 2017), and achieved the best performance with POS and dictionary features (F1-measure of 86.11% in CCKS 2018, F1-measure of 90.48% in CCKS 2017). In particular, the BiLSTM-Att-CRF model had significant effect on the improvement of Recall.

**Conclusions:**

Our work preliminarily confirmed the validity of attention mechanism in discovering key information and mining text features, which might provide useful ideas for future research in clinical named entity recognition of Chinese electronic medical records. In the future, we will explore the deeper application of attention mechanism in neural network.

## Background

Electronic medical records (EMRs) contain rich health data and important clinical evidence, which are helpful to support clinical decision-making and disease monitoring [1]. But the large number of unstructured clinical texts limit the large-scale knowledge discovery and application of electronic medical records [2]. It is urgent to explore the auto-information extraction methods to transform unstructured texts into structured data that are easy to understand and use for computers.

As a key step in natural language processing (NLP), clinical named entity recognition (CNER) has been a popular research topic on extracting all kinds of meaningful information in unstructured clinical text. Early studies focused on designing characteristic templates with the help of linguistic knowledge and professional dictionaries [3]. With the publication and application of large-scale electronic medical record corpus [4], the method of named entity recognition based on statistical learning has been widely used and proven to achieve good performance in many studies [5, 6]. But traditional machine learning methods depend on large tagged corpus and effective feature engineering. In order to reduce the dependence on linguistic knowledge and complicated feature engineering, deep learning with muti-layer neural network structure has become the most popular method for clinical entity recognition [7–9].

The clinical text features of Chinese electronic medical records pose many challenges to named entity recognition task. Firstly, as shown in the examples of Table 1, the clinical texts are more objective than the common natural language, and the logic of semantic is relatively concentrated. Therefore, it is difficult to reuse the common domain language model in the named entity recognition of electronic medical records. Secondly, there are so many professional and uncommon clinical terms used to describe different patients’ situations. And many terms are expressed in the different form of English abbreviations or another names, such as “直肠低位前切除术 (low anterior resection)” is also expressed as “Dixon术 (Dixon operation)” in the same clinical text. Different expressions of clinical terms make it difficult to use Chinese medical dictionary for effective entity recognition. In addition, the use of Chinese word is more flexible, sometimes we can’t judge whether the word is a named entity in the context, and even if it is a named entity, it may belong to different types in different context. For example, “双侧输卵管切除术 (bilateral salpingectomy)” should be recognized as an “Operation” entity as a whole, but “双侧输卵管 (Bilateral tubal)”should be recognized as an “Anatomical” entity when it appears alone. In this case, the method of clinical named entity recognition needs strong learning ability to capture the key context-critical information, which can improve the recognition performance both on entity type and entity boundary.

**Table 1:** Example of Chinese EMRs

Chinese electronic medical record text	
缘于入院前20余日于我院诊为乙状结肠癌, 在全麻下行乙状结肠癌根治术, 术中见:腹腔内无明显腹水, 腹腔、盆腔、大网膜无明显转移结节, 肝脏质地大小正常, 未触及肿物, 胆囊未触及结石。 More than 20 days before hospitalization, the patient was diagnosed with sigmoid colon cancer in our hospital and radical resection of sigmoid colon cancer was performed under general anesthesia. Intraoperative findings: No obvious ascites was found in abdominal cavity; no obvious metastatic nodules were found in abdominal cavity, pelvic cavity or omentum; the texture and size of liver were normal and no tumors were touched; no gallbladder stone was touched.	

In most previous studies, Chinese word embedding and traditional domain features were used to improve the performance of deep learning model in CNER tasks. In the China Conference on Knowledge Graph and Semantic Computing (CCKS) 2018 CNER challenge [10], the team from Alibaba Health Information Technology used cw2vec method to construct word embedding for the first time in the field of medical texts [11], they explored the features contained in strokes and radicals of Chinese characters and finally won the first place with an F1-measure of 89.13% [12]. Zhang et al. added 50-dimensional word embedding, 50-dimensional dictionary embedding and stroke embedding trained by cw2vec tool as additional features to the training of neural network models and finally achieved good experimental results (F1-measure of 88.64%) [13]. Although these methods are effective, they also spend a lot of time on selecting features and training word embedding.

Attention mechanism was initially used in the field of image recognition to emphasize the different influence of different input data on output data [14]. It is a selective mechanism for allocating information processing capabilities, which can selectively focus on some important information and ignore other information received at the same time. In recent two years, it has been widely used in sequential learning tasks of NLP and achieved excellent performance [15, 16]. Sui proposed an Encoder-Decoder-CRF model with Attention mechanism to solve the problem of redundancy or missing of context information in Chinese NER tasks, results showed the model with attention mechanism performed better in the recognition of organization and place names than Bi-LSTM-CRF model, thus it could be more flexible to obtain appropriate context information for entities [17]. Ma et al. proposed an attention-based BLSTM-CRF model for new energy vehicle patent terminology extraction and corrected the results using a dictionary-based and rule-based method. The accuracy of this model reached more than 86% [18]. Luo et al. proposed a novel attention-based BiLSTM-CRF approach for document-level chemical NER to solve the tagging inconsistency problem, finally achieved the state-of-the-art performances on the BioCreative IV CHEMDNER corpus and the BioCreative V chemical-disease relation (CDR) corpus [19].

Over all, the attention mechanism can make up for the shortcomings of the semantic representation in the traditional encoder-decoder model, and it is easier to capture the long-distance interdependent features in the sentence [20]. Therefore, aiming to improve the performance of clinical named entity recognition in Chinese EMRs, we proposed a deep learning model named BiLSTM-Att-CRF that combined bidirectional long-short time memory network with attention mechanism.

## Methods

### Data preprocessing

**Fig. 1:**
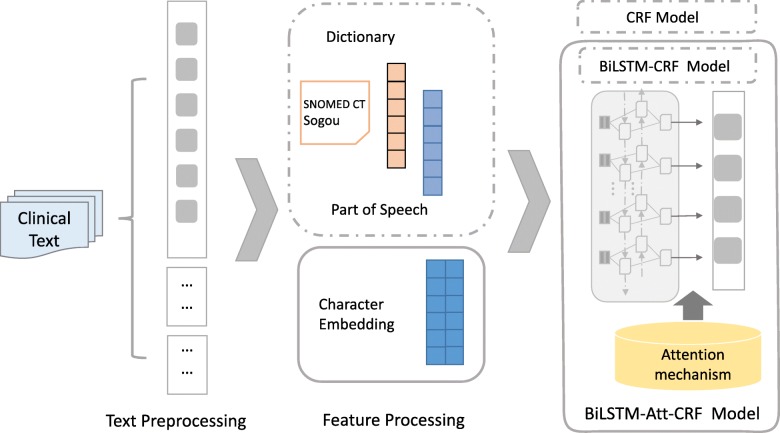
The processing flow of our method

The processing flow of our method is shown in Fig. 1. Firstly, some preprocessing steps including data annotation, sentence splitting and character segmentation were performed. In order to preserve the meaning of the special punctuation marks in the clinical text, e.g. “CERBB-2(2+)”, and considering the centralized semantic logic of clinical texts, we split the input sentences by commas and periods. Unlike English, Chinese clinical texts don’t have space as the boundary mark of words, and there are many different combinations of characters. In order to avoid the entity boundary recognition errors caused by word segmentation, we took characters as the input of the model.

Recently, known as distributed feature representation, word embedding has been widely used in the field of natural language processing, especially for deep learning methods. Compared with the one-hot representation based on the bag-of-words (BOW) and n-gram method, word embedding is low dimensional and dense,it can automatically measure the semantic similarity of words from a large unlabeled corpus [21]. In recent years, several tools such as word2vec [22] and GloVe [23] have been widely used in the field of NLP. Consistent with character-based input, we employed the character embedding as the basic feature. To achieve a high-quality pre-trained character embedding, a total of 3605 medical records were collected from the CCKS 2017 [24] and CCKS 2018 CNER challenge task [10], they were all used to train 200-dimensional character embedding by the word2vec tool.

Clinical texts usually have relatively fixed syntax and common expressions, therefore traditional linguistic and domain features based on dictionaries and grammatical structures can effectively improve the performance of CNER [25]. So additional features including dictionary and part-of-speech were introduced into this study. Systematized Nomenclature of Medicine-Clinical Terms (SNOMED-CT) is the most comprehensive clinical terminology covering 19 clinical categories. We built our dictionaries based on the terms from three categories (“Body structure”, “Procedure”, “Pharmaceutical/biologic product”) related to our NER task. We also collected the dictionaries from Sogou to supplement more commonly used medical terms to our dictionaries, including “Generic list of commonly prescribed drugs”, “Surgical classification code (ICD-9-CM3)“and “Human anatomy” [26]. After removing duplicates and irrelevant words, finally three dictionaries (named Dic_anatomy, Dic_drug, and Dic_operation) were built. The details of each dictionary are shown in Table 2. Bi-direction maximum matching method was used to capture longest possible match, and each character in the match was encoded in “B/I/O/E/S+ dictionary name” tagging scheme. Furthermore, the character-level part of speech (POS) tags were generated by Jieba segmentation system [27]. At last, the lookup table was used to output 100-dimensional additional feature embedding.

**Table 2:** Details of custom dictionaries

	Source	Number
Dic_anatomy	SNOMED CT: Body structure Sogou: Human anatomy	6401
Dic_drug	SNOMED CT: Pharmaceutical/biologic product Sogou: Generic list of commonly prescribed drugs	35,151
Dic_operation	SNOMED CT: Procedure Sogou: Surgical classification code (ICD-9-CM3)	5840

### BiLSTM-Att-CRF model

**Fig. 2:**
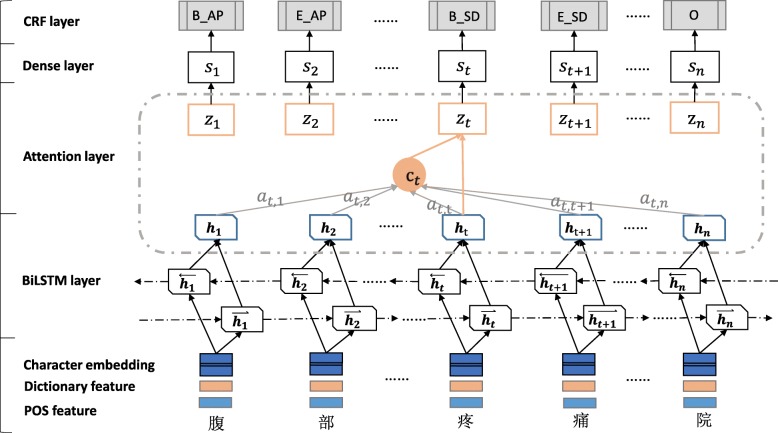
The structure of BiLSTM-Att-CRF model

We proposed an improved deep learning model named BiLSTM-Att-CRF which applied the attention mechanism to the basic BiLSTM-CRF model. The framework of BiLSTM-Att-CRF model is illustrated in Fig. 2.

As a sequence-labeling task, the model determined a sequence of labels with the largest joint probability for the sequence of input tokens and a predefined set of labels. In this paper, we utilized the “BIESO” (Begin, Inter, End, Single, Other) tagging scheme to represent the position of the tokens within the entities. The first layer of model is embedding layer, by looking up the pre-trained character embedding table, the input sentence is represented as a sequence of vectors X = (X_1_, X_2_, X_3,_…, X_*n*_), where n was the length of the sentence. Next, the vectors are given as the input to the BiLSTM layer.

LSTM (Long Short-Term Memory) is a special form of traditional recurrent neural network (RNN). By introducing memory cell, LSTM network can effectively utilize more information and solve the gradient vanishing or exploding problems of RNN [28]. The hidden layer of LSTM network is composed of specially constructed memory cells. Each memory cell learns the context characteristics of input text under the synergistic action of loop connection cell, input gate, output gate and forget gate. Two separate hidden states are always used to capture both past (forward) and future (backward) context information. In our BiLSTM (Bidirectional Long Short-Term Memory) layer, a forward LSTM computed a representation $\overset{\rightharpoonup }{\mathrm{H}}=\left({\overrightarrow{\mathrm{h}}}_1,{\overrightarrow{h}}_2,{\overrightarrow{h}}_{3,}\dots, {\overrightarrow{h}}_n\right)$ the sequence from left and another backward LSTM computed a representation of $\overleftarrow{\mathrm{H}}=\left({\overleftarrow{\mathrm{h}}}_1,{\overleftarrow{h}}_2,{\overleftarrow{h}}_{3,}\dots, {\overleftarrow{h}}_n\right)$ in reverse. For every word, ${\mathrm{h}}_t=\left[{\overrightarrow{\mathrm{h}}}_t,{\overleftarrow{h}}_t\right]$ was represented by concatenating two distinct context representations.

Different from the traditional BiLSTM-CRF model put the out of BiLSTM layer as the final feature directly, we added an attention layer on the top of the BiLSTM layer to capture more contextual feature in the sentence. Value *α*_tj_ in the attention matrix is the attention weigh which computed by comparing the current target word representation x_t_ with other word x_j_ in the same sentence:

1 $$ {\alpha}_{\mathrm{tj}}=\frac{\exp \left({e}_{tj}\right)}{\sum_{k=1}^n\exp \left({e}_{tk}\right)} $$

Here, e_tj_ is usually a single-layer or multi-layer perceptron, which is called alignment function, the values will be larger with the increase of similarity. Where the weigh *W*_*a*_ needs to be learned in the process of training.

2 $$ {\mathrm{e}}_{\mathrm{tj}}=\tanh \left({W}_a\left[{x}_t;{x}_j\right]\right) $$

Then a global vector *c*_*t*_ is computed as a weighted sum of each BiLSTM output *h*_*j*_:

3 $$ {c}_t={\sum}_{j=1}^n{\alpha}_{tj}{h}_j $$

Next, we concatenated the global vector *c*_*t*_ and the BiLSTM output *h*_*t*_ *into* a vector z_t_ to represent each word, the vector is fed to a tanh function to produce the output of attention layer.

4 $$ {\mathrm{z}}_{\mathrm{t}}=\tanh \left({W}_z\left[{c}_t;{h}_t\right]\right) $$

Then, through the Dense layer, the decision probability of s_t_ mapping to the annotated result is expressed as.

5 $$ {\mathrm{s}}_{\mathrm{t}}=\tanh \left({W}_p{z}_t\right) $$

Finally, instead of modeling tagging decisions independently, the CRF layer is added to decode the best tag path in all possible tag paths. In the CRF layer, the current input is predicted by the past input and the state to which the input belongs, the score by moving from state i to state j is represented by the probability transfer matrix T_i, j_, the element P_i, j_ of the matrix is the score of the j^th^ tag of the i^th^ word in the sentence. The maximum likelihood estimation is used as the loss function, and the Viterbi algorithm is used to compute optimal tag sequences for inference. The calculation formula of the output state sequence Y = (y_1_, y_2_, y_3,_…, y_*n*_) is as:

6 $$ \mathrm{S}\left(\mathrm{X},\mathrm{Y}\right)={\sum}_{i=0}^n{T}_{y_i,{y}_{i+1}}+{\sum}_{i=1}^n{P}_{i,{y}_i} $$

7 $$ \log \left(P\left(Y|x\right)\right)=S\left(X,Y\right)-\log {\sum}_{y\hbox{'}}{e}^{S\left(X,{y}^{\hbox{'}}\right)} $$

## Result

### Dataset

In this study, Two Chinese EMRs datasets released by CCKS CNER challenge were used to train our model [10]. The distribution of entities in two datasets is shown in Table 3, we will mainly discuss the results on the CCKS 2018 dataset in this study. A total of 1000 records from CCKS 2018 are officially divided into 600 training data and 400 test data, and five categories of entity are pre-defined: (1) Anatomical Part (AP), the functional structural unit of body, such as “腹部 (abdomen)”; (2) Symptom Description (SD), patient’s abnormal experience or feeling that needs to be combined with the anatomical part, such as “不适 (uncomfortable)”; (3) Independent Symptom (IS), patient’s abnormal experience and feeling that can be independently output, such as “呕吐 (emesis)”; (4) Drug, a chemical substance used in the treatment of disease to enhance physical or mental well-being, such as “阿莫西林 (Amoxil)”; (5) Operation, the medical treatment of injuries or diseases, such as “直肠癌根治术 (colorectal tumor surgery)”. Another 400 records were derived from CCKS 2017 CNER challenge which focus on the named entity recognition of “Disease”, “Symptom”, “Treatment”, “Test” and “Anatomical Part”. Each entity in our datasets is annotated as {entity, start position, end position, entity type}, the example is shown in Table 4.

**Table 3:** Distribution of entities in two datasets

	Dataset of CCKS 2018			Dataset of CCKS 2017	
	Training set (600)	Test set (400)	Entity type	Training set (300)	Test set (100)
Anatomical Part	7838 (52%)	6339 (63%)	Body Part	10,719 (36%)	3021 (32%)
Symptom Description	2066 (14%)	918 (9%)	Symptom	7831 (26%)	2311 (24%)
Independent Symptom	3055 (20%)	1327 (13%)	Diagnosis	722 (2%)	553 (6%)
Drug	1005 (7%)	813 (8%)	Test	9546 (32%)	3143 (33%)
Operation	1116 (7%)	735 (7%)	Treatment	1048 (4%)	465 (5%)

**Table 4:** Example of the manually annotated records

Clinical text: 患者1个月前无明显诱因出现上腹部不适 … (The patient had no obvious inducement of upper abdominal uncomfortable one month ago …)			
Entity	Start Position	End Position	Entity Type
上腹部 (upper abdomen)	13	15	Anatomy
不适 (uncomfortable)	16	17	Symptom

### Experimental settings

Our deep learning models were implemented using open-source library Tensorflow and Keras for Python 3.6, Table 5 shows the adopted hyper-parameters in our study. CRF++(0.58) tool for python was used to train our basic CRF model. We fixed the content window size at 5 and built 33 Unigram templates to extracting context character.

**Table 5:** Hyper-parameters of deep learning models

Parameter	Value
Character embedding size	200
Additional features embedding size	100
Maximum training Epoch	30
Batch size	32
Time steps	150
Learning rate	0.001
Size of LSTM hidden units	300
Dropout rate	0.2
Optimizer	Adam

### Evaluation metrics

According to the evaluation metrics provided by CCKS 2018 CNER task organizer [10], “Strict” metrics was defined as a correct match that the ground truth and the mention shared same mention, same boundaries (start position, end position) and same entity type. Precision (P), Recall (R), and F1-measure were used in our experiments to evaluate the recognition performance under the “Strict” metrics.

### Evaluation results

In order to verify the effectiveness of the attention-based deep learning model in the CNER task of Chinese electronic medical record, the basic CRF model and BiLSTM-CRF model which had achieved good performance in previous studies were selected as comparative experiments. In this chapter, we compared the recognition performance of the basic models on different types of entities, and analyzed the influence of additional features.

#### Performance comparison of BiLSTM-Att-CRF model and basic models

By comparing the results of three basic models shown in Table 6, our BiLSTM-Att-CRF model achieves better performance than BiLSTM-CRF model and CRF model in two datasets. Without adding other external resources and additional features, two deep learning models are more effective than the traditional CRF model. They can not only learn the similarity between input characters by the pre-trained character embedding, but also capture more context information through the units in LSTM layer. But in the CRF model, limited context information can be learned within the fixed window. Moreover, attention layer added in the BiLSTM-Att-CRF model can learn the structure of sentences directly and capture the relationships between two tokens regardless of their distance. The contribution of attention mechanism will be discussed in details in the next section.

**Table 6:** Performance comparison of BiLSTM-Att-CRF model and basic models

	Dataset of CCKS 2018			Dataset of CCKS 2017		
	Precision	Recall	F-score	Precision	Recall	F-score
CRF	85.63%	79.58%	82.49%	87.32%	83.06%	85.14%
+POS	85.94%	81.04%	83.42%	88.97%	85.12%	87.01%
+Dic	**88.98%**	82.82%	85.79%	90.34%	86.06%	88.15%
+POS + Dic	88.72%	83.57%	86.04%	**91.26%**	87.11%	89.14%
BiLSTM-CRF	85.20%	83.09%	84.13%	90.09%	89.24%	89.66%
+POS	85.23%	82.73%	83.96%	89.36%	89.74%	89.55%
+Dic	85.62%	83.34%	84.46%	90.17%	90.44%	90.30%
+POS + Dic	86.02%	82.93%	84.45%	90.31%	90.12%	90.22%
BiLSTM-Att-CRF	86.51%	84.38%	85.43%	90.11%	90.47%	90.29%
+POS	86.62%	84.36%	85.48%	90.33%	89.88%	90.10%
+Dic	86.97%	84.79%	85.87%	89.87%	**90.75%**	90.31%
+POS + Dic	87.09%	**85.13%**	**86.11%**	90.41%	90.49%	**90.48%**

The bold values denote the highest values

**Fig. 3:**
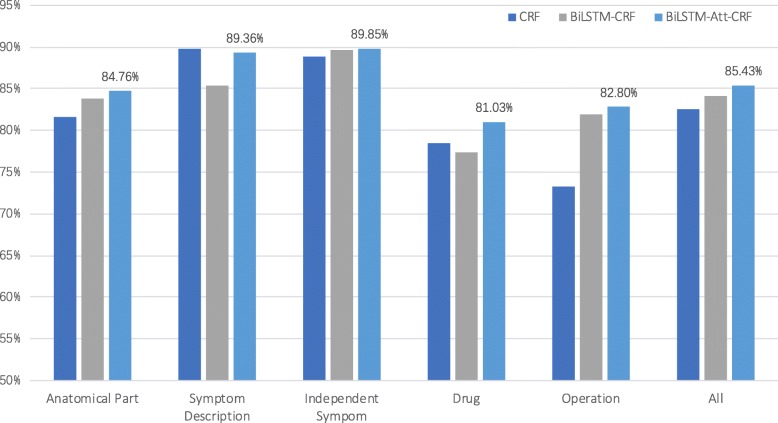
Recognition performance of five entity types in CCKS 2018 dataset

As shown in Fig. 3, we analyzed entity recognition performance on five types in CCKS 2018 dataset. The BiLSTM-Att-CRF model achieves better performance on most types of entities, but it is a little worse on “Symptom Description” entities than CRF model. The limitation of dataset is one possible reason. There are a lot of “Symptom Description” entities with inconsistent labels in the training set, for example, “不适 (uncomfortable)” is annotated as “Independent Symptom” in the context of “进食不适 (eating was uncomfortable)”, it is also annotated as “Symptom Description” in the context of “上腹胀痛不适 (abdominal was pain and uncomfortable)”, but it is not annotated as any type in “无其他不适”(no other uncomfortable). Furthermore, the semantically related information of “Symptom Description” entities usually have longer distance from the entities, and sometimes they might not be in one sentence split by commas. Therefore they cannot be learned by our sentence-level attention layer, such as the sentence of “主诉14天前患者出现中下腹部闷痛不适|间歇性痛|翻身向右侧时疼痛有所缓解 (The patient complained of mid-lower abdominal pain and discomfort 14 days ago| intermittent pain| when turning over to the right, the pain was relieved)” . The attention-based model could not capture the key information “中下腹部 (mid-lower abdominal)”, so “间歇性痛 (intermittent pain)” and “疼痛 (pain)” were incorrectly recognized as “Independent Symptom” . The recognition performance on “Drug” entities was also not as expected without adding the dictionary feature. Except for the small size of the training samples, we speculated that the large number of obscure words and abbreviations in “Drug” entities may interfere with the recognition effect, for example, “调整方案TXT+DDP*3(Adjustment scheme TXT+DDP*3)”. Moreover, there are many unprecedented “Drug” entities in the test set, so the ability of BiLSTM-Att-CRF model to discover and recognize new words needs to be improved by combining large medical knowledge base.

#### The effect of additional features

We also analyzed the effect of two additional features (POS, dictionary) on the performance of our models. As shown in Table 6, the BiLSTM-Att-CRF model achieves the best performance by adding additional features (F1-measure of 86.11% in CCKS 2018 and 90.48% in CCKS 2018). The P and R value of CRF model are greatly improved by adding additional features, it proves that the traditional linguistic and domain features can help to improve the performance of the statistical learning model. Particularly, dictionary feature contributed more than POS. However, the two neural network models have limited improvement after adding additional features, and the results are even worse than baseline models when add POS only. Although the custom dictionary was used in POS tagging process, there were still many boundary errors of nested clinical terms, such as “行/v广/a泛/a性/a子/n宫/n切/v除/v +/x双/m侧/v输/n卵/n管/n卵/n巢/n切/v除/v术/n”. In the previous study, Cai et al. proposed a Reduced-POS tagging method to improve the accuracy of Chinese entity boundary detection [29]. In the future, more effective features pre-trained by deep learning model should be chosen to enhance the recognition performance of the neural network model.

## Discussion

### Performance of BiLSTM-Att-CRF model in improving Recall

Higher Recall means that the model can memorize more details related to entities and classify more unrecognized or misjudged entities into correct entity types. Table 7 shows the Recall among three baseline models without additional features in CCKS 2018 dataset, we can see that Recall is improved significantly in our BiLSTM-Att-CRF model, especially in types of “Operation” and “Drug” entities which always difficult to recognize. Compared to other types of entities, “Operation” entities usually have long length and contain nested structures, which easily cause boundary errors of recognition. But there is also a good point, some fixed keywords are always used around “Operation” entities, e.g. “行 (undergo)”, “术 (surgery)”. As the example of recognition performance shown in Table 8, all three models can recognize the entity when the keyword is closed to it in the context, but CRF model can’t capture context information of a little bit long-distance. Better than the CRF model, LSTM can solve hard long-time lag problems with the gating mechanism, but later words are more dominant than earlier words, which leads to recognition difficulty on long sentences. Therefore, the attention mechanism performs better when the keyword is long away or the length of entity is too long.

**Table 7:** Recall of Bilstm-Att-CRF model and other basic models

	CRF	BiLSTM-CRF	BiLSTM-Att-CRF
Anatomical Part	80.88%	83.55%	**84.24%**
Symptom Description	86.06%	84.22%	**87.70%**
Independent Symptom	83.33%	88.78%	**89.61%**
Drug	69.25%	71.74%	**75.31%**
Operation	64.90%	80.05%	**81.95%**

The bold values denote the highest values

**Table 8:** Example of recognition performance of Bilstm-Att-CRF model and other basic models

	CRF	BiLSTM-CRF	BiLSTM-Att-CRF
行乙状结肠癌根治术 (undergo radical resection of sigmoid colon cancer)	OP:乙状结肠癌根治术^*^ (radical resection of sigmoid colon cancer)	OP:乙状结肠癌根治术^*^ (radical resection of sigmoid colon cancer)	OP:乙状结肠癌根治术^*^ (radical resection of sigmoid colon cancer)
行局麻下区域淋巴结切除术 (undergo regional lymphadenectomy under local anesthesia)	OP:淋巴结切除术 (lymphadenectomy)	OP:局麻下区域淋巴结切除术 (regional lymphadenectomy under local anesthesia)	OP:区域淋巴结切除术^*^ (regional lymphadenectomy)
行胰腺肿瘤射频消融+无水酒精注射+胆总管十二指肠吻合+胃空肠吻合+胆囊切除术 (undergo radiofrequency ablation of pancreatic tumors+ethanol injection+ choledochoduodenostomy + gastrojejunostomy + cholecystectomy)	AP: 胰腺 (pancreas) Drug: 酒精 (ethanol) AP: 胆总管十二指肠 (common bile duct and duodenal) OP: 胆囊切除术^*^ (cholecystectomy)	OP:胰腺肿瘤射频消融^*^ (radiofrequency ablation of pancreatic tumors) AP:胃空肠 (gastrojejunal) OP:胆囊切除术^*^ (cholecystectomy)	OP:胰腺肿瘤射频消融^*^ (radiofrequency ablation of pancreatic tumors) OP:胆总管十二指肠吻合^*^ (choledochoduodenostomy) OP:无水酒精注射^*^ (ethanol injection) OP:胃空肠吻合^*^ (gastrojejunostomy) OP:胆囊切除术^*^ (cholecystectomy)
术后行2周期紫衫+DDP化疗(2 cycles chemotherapy of taxol +DDP after the operation)	__	OP: DDP化疗 (DDP chemotherapy)	Drug:紫衫^*^ (taxol) Drug: DDP^*^

The * denote the correct recognition. *OP* Operation, *AP* Anatomical Part

### Performance of different attention widths

**Fig. 4:**
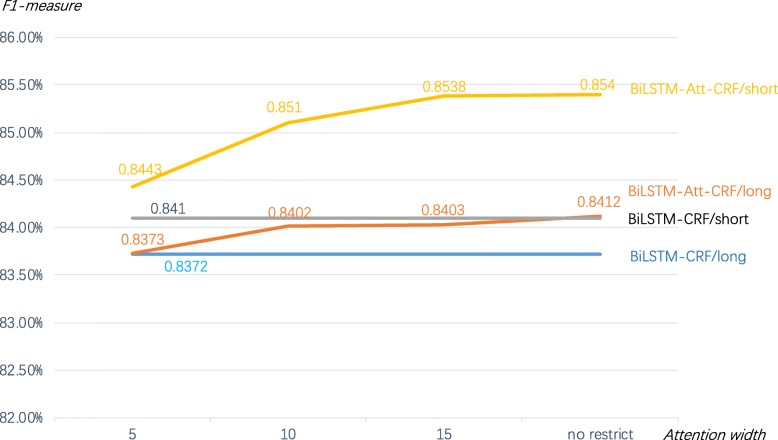
Recognition performance of different attention widths

Not all problems need long-term or globally dependent attention mechanism, many problems only rely on local features [14]. As introducted in Background, the semantic logic of clinical text is relatively concentrated and related semantic information are basically concentrated in one short sentence. In order to explore the influence of different attention widths on named entitity recognition of Chinese NERs, we added different attention widths in the training process based on the Bilstm-Att-CRF model. As shown in Fig. 4, the attention width *r* means that the current word is only associated with the *r* words before and after it, so attention only be computed between the (2*r* + 1) words, “long” means the input sentence of model was split only by period (“。”, length: Ave =48, Max = 455), and “short” means the input sentence was split by comma and period (“,” & “。”, length: Ave = 15, Max = 176). The results show that F1-measure improves with the increase of attention width regadless of whether long or short input, but when attention width increases to a large value, the F1-measure improves slowly. And the F1-measure of short input performe better than that of long input. So, we found that the meaning of each short sentence in the Chinese clinical text is relatively independent, and the key information does not need to be learned from too long distance, local feature learning with proper attention width could achieve good performance in the clinical entity recognition of Chinese EMRs.

## Conclusion

In this paper, we integrated the attention mechanism into neural network, and proposed an improved clinical named entity recognition method for Chinese electronic medical records called BiLSTM-Att-CRF. Recognition Performance showed that this model could capture more useful information of context and avoided the problem of missing information caused by long-distance factors, especially performed in Recall improvement. In the CNER task of CCKS 2018 and CCKS 2017, our BiLSTM-Att-CRF model finally achieved better performance than other widely-used models without additional features(F1-measure of 85.4% in CCKS 2018, F1-measure of 90.29% in CCKS 2017), and achieved the best performance with POS and dictionary features (F1-measure of 86.11% in CCKS 2018, F1-measure of 90.48% in CCKS 2017), which also verified the validity of additional features. Moreover, the influence of different attention widths in BiLSTM-Att-CRF model was also discussed, which indicated that choosing appropriate attention width to focus on local feature learning can achieve good performance. Overall, this paper preliminarily confirmed the effectiveness of attention mechanism in the field of clinical named entity recognition, which proved some useful ideas for future research in this field.

However, there are still some shortcomings in our study. The BiLSTM-Att-CRF model has insufficient ability to recognize new words, and the selection and generation of pre-trained feature embedding should be more careful. In order to achieve better recognition performance, our future research will explore the deeper application of attention mechanism in neural network, such as the “BERT” model proposed by Google AI Language team [30].

## Data Availability

The datasets used in this study are adopted from the Chinese EMR named entity recognition task in China Conference on Knowledge Graph and Semantic Computing in 2018 (http://www.ccks2018.cn/) and 2017(http:// www.ccks2017.cn/), but restrictions apply to the availability of these data, which were used under license for the current study, and so are not publicly available.

## Abbreviations

AP: Anatomical Part
BiLSTM: Bidirectional Long Short-Term Memory
BOW: Bag-of-words
CCKS: China Conference on Knowledge Graph and Semantic Computing
CNER: Clinical named entity recognition
CNN: Convolutional Neural Network
CRF: Conditional Random Field
EMRs: Electronic medical records
IS: Independent Symptom
LSTM: Long Short-Term Memory
P: Precision
POS: Part-of-speech
R: Recall
RNN: Recurrent Neural Network
SD: Symptom Description
SNOMED-CT: Systematized Nomenclature of Medicine-Clinical Terms

## References

[1] Lossio-Ventura JA, Hogan W, Modave F, Hicks A, Hanna J, Guo Y, et al. Towards an obesity-Cancer Knowledge Base: biomedical entity identification and relation detection. IEEE International Conference on Bioinformatics and Biomedicine. 2016. p. 1081–8. 10.1109/BIBM.2016.7822672 PMC5426361 28503356

[2] Jensen PB, Jensen LJ, Brunak S (2012). Mining electronic health records: towards better research applications and clinical care. Nat Rev Genet.

[3] Friedman C, Alderson PO, Austin JHM, Cimino JJ, Johnson SB (1994). A general natural-language text processor for clinical radiology. J Am Med Inform Assn.

[4] i2b2. i2b2: Informatics for Integrating Biology & the Bedside. https://www.i2b2.org/NLP/DataSets/Main.php. Date Accessed: 3/25/2019.

[5] Liu KX, Hu QC, Liu JW, Xing CX. Named Entity Recognition in Chinese Electronic Medical Records Based on CRF. 2017 14th Web Information Systems and Applications Conference (Wisa 2017). 2017:105–110.

[6] Tang BZ, Cao HX, Wu YH, Jiang M, Xu H. Clinical entity recognition using structural support vector machines with rich features. Proceedings of the Acm Sixth International Workshop on Data and Text Mining in Biomedical Informatics. 2012. p. 13–9.

[7] Wu YH, Jiang M, Lei JB, Xu H (2015). Named entity recognition in Chinese clinical text using deep neural network. Stud Health Technol.

[8] Zeng D (2017). LSTM-CRF for drug-named entity recognition. Entropy..

[9] Chalapathy R, Borzeshi EZ, Piccardi MJ. Bidirectional LSTM-CRF for clinical concept extraction. Proceedings of COLING. 2016.

[10] CCKS 2018. China Conference on Knowledge Graph and Semantic Computing 2018. http://www.ccks2018.cn. Date Accessed: 12/21/2018.

[11] Cao, et al. cw2vec: learning Chinese word embeddings with stroke n-gram information. Proceedings of the 32th AAAI Conference on Artificial Intelligence. 2018.

[12] Tmtpost. With the cw2vec method, Alibaba Health won the national champion of Chinese electronic medical record entity recognition. http://www.tmtpost.com/nictation/3424059.html. Date Accessed: 3/22/2019.

[13] Luo L, Li N, Li S, Yang Z. DUTIR at the CCKS-2018 Task1: A Neural Network Ensemble Approach for Chinese Clinical Named Entity Recognition. Proceedings of the Evaluation Tasks at the China Conference on Knowledge Graph and Semantic Computing (CCKS 2018). 2018. p. 7-12.

[14] Vaswani A, Shazeer N, Parmar N, Uszkoreit J, Jones L, Gomez AN, et al. Attention Is All You Need. arXiv:1706.03762 [cs.CV]. https://arxiv.org/abs/1706.03762.

[15] Bahdanau D, Cho K, Bengio YJ. Neural machine translation by jointly learning to align and translate. Proceedings of International Conference on Learning Representations. 2014.

[16] Tan Z, Wang M, Xie J, Chen Y, Shi X, editors. Deep semantic role labeling with self-attention. Thirty-Second AAAI Conference on Artificial Intelligence. 2018.

[17] Sui C (2017). Research of Chinese named entity recognition based on deep learning.

[18] Ma J, Zhang Y, Yao S, et al. Terminology extraction for new energy vehicle based on BiLSTM_Attention_CRF model. Application Research of Computers. 2019;36(05):1385-9.

[19] Luo L, Yang Z, Yang P, Zhang Y, Wang L, Lin H (2018). An attention-based BiLSTM-CRF approach to document-level chemical named entity recognition. Bioinformatics..

[20] Rui Z, Wang Z, Mai D (2017). Building Emotional Conversation Systems Using Multi-task Seq2Seq Learning. Natural Language Processing and Chinese Computing.

[21] Lai SW, Liu K, He SZ, Zhao J (2016). How to generate a good word embedding. IEEE Intell Syst.

[22] Mikolov T (2013). Distributed representations of words and phrases and their compositionality. Adv Neural Inf Process Syst.

[23] Pennington J, Socher R, Manning C, editors. Glove: Global vectors for word representation. Proceedings of the 2014 conference on empirical methods in natural language processing (EMNLP). 2014.

[24] CCKS 2017. China Conference on Knowledge Graph and Semantic Computing 2017. http://www.ccks2017.cn. Date Accessed: 3/25/2019.

[25] Wang Q, Zhou Y, Ruan T (2019). Incorporating dictionaries into deep neural networks for the Chinese clinical named entity recognition. J Biomed Inform.

[26] Sogou pinyin. Sogou Dict. https://pinyin.sogou.com/dict. Date Accessed: 3/25/2019.

[27] Jieba. Chinese Words Segementation Utilities. https://pypi.org/project/jieba. Date Accessed: 3/25/2019.

[28] Jozefowicz R, Zaremba W, Sutskever I, editors. An empirical exploration of recurrent network architectures. International Conference on International Conference on Machine Learning. 2015.

[29] Cai X, Dong S, Hu J (2019). A deep learning model incorporating part of speech and self-matching attention for named entity recognition of Chinese electronic medical records. BMC Med Inform Decis Making.

[30] Devlin J, Chang MW, Lee K, et al. Bert: Pre-training of deep bidirectional transformers for language understanding. arXiv:1810.04805 [cs.CL]. https://arxiv.org/abs/1810.04805.

